# Issue at a glance

**DOI:** 10.1093/ehjimp/qyae090

**Published:** 2024-10-07

**Authors:** Oliver Gaemperli

**Affiliations:** HeartClinic Hirslanden, Witellikerstrasse 40, Zurich CH-8053, Switzerland

Valvular heart disease (VHD) is a significant cause of disability, morbidity, and reductions in quality of life and longevity. In low- to middle-income countries, rheumatic heart disease remains the predominant VHD aetiology affecting ∼41 million people worldwide. In contrast, calcific aortic stenosis and degenerative mitral valve disease are more common in higher-income countries, and affect 9 and 24 million people, respectively. In hospitalized patients with heart failure (HF), significant VHD is found in approximately one-third of patients, either contributing to or directly causing cardiac congestion. Despite reductions in global mortality from rheumatic heart disease over the last century, death rates have remained otherwise relatively stable. In fact, the global trend to overaging has resulted in a significant and ongoing rise in mortality for calcific aortic stenosis since the 2000s.

The last decades have witnessed massive improvements in diagnostic approaches and therapeutic options for the large population of patients suffering from VHD. On the one hand, technical improvements in echocardiographic techniques, multimodality imaging with incorporation of computed tomography, magnetic resonance and radionuclide imaging into diagnostic work-ups, and lately introduction of artificial intelligence have improved detection, grading, and phenotyping of VHD. On the other hand, refinement of surgical techniques, the introduction of transcatheter therapies, and the consolidation of valvular heart teams (debating therapeutic choices among expert cardiologist, surgeons and anaesthetists) have created a new dynamic approach to treatment of VHD. The role of imaging methods is central in this process, allowing accurate diagnosis, guiding surgical or transcatheter procedures, and providing reliable follow-up of VHD patients. It is with this in mind that the editorial board of the *European Heart Journal – Imaging Methods and Practice* felt compelled to devote an entire Special Issue to this important and dynamic topic of VHD.

More than 9 million patients suffer from calcific aortic stenosis worldwide. Due to advanced age and comorbidities, between 30% and 50% of patients are rejected by surgeons fearing excessive surgical risk. The wide introduction of transcatheter aortic valve implantation (TAVI) in the late 2000s has been a game-changer offering effective treatment to patients who were otherwise denied surgery. On the other hand, up to 6% of patients presenting for aortic valve replacement have bicuspid aortic valves (BAV). Generally, BAV patients develop valve failure earlier in life than their tricuspid aortic valve counterparts. However, cardiac mechanics and the ideal timepoint of intervention remain poorly understood. In the Special Issue on Valvular heart disease, Keuning *et al*.^1^ analysed left ventricular (LV) strain–volume loops derived from transthoracic echocardiography in 113 patients with BAV and compared them with age- and sex-matched controls. BAV patients demonstrated lower Sslope (linear slope of the strain–volume relation during systole) (0.21%/mL vs. 0.27%/mL, *P* < 0.001) and ESslope (early linear slope during the first 5% of volume change during systole) (0.19%/mL vs. 0.29%/mL, *P* < 0.001) compared with controls, but also greater uncoupling (mean difference between systolic and diastolic strain for any given volume) during early (0.48 vs. 0.05, *P* = 0.04) and late diastole (0.66 vs. −0.07, *P* < 0.001). Over a follow-up period of 10 years, peak aortic jet velocity, enlarged left atrium (LA), E/e′ ratio, global longitudinal strain, and ESslope emerged as significant and independent predictors of cardiac events (mainly arrhythmia and/or aortic valve replacement). The study provides insights into altered LV cardiomechanics of BAV patients and suggests a significant impact of strain–volume relationships (particularly during early systole) on outcomes.

Paravalvular aortic regurgitation (PAR) after TAVI remains an important issue associated with late post-operative morbidity and mortality. In their review of literature, van Wely *et al*.^2^ provide an overview on incidence, detection and quantification methods, and prognostic impact of PAR: new-onset significant PAR after TAVI has detrimental effects on the non-compliant LV’s of patients with severe aortic stenosis, resulting in disproportionally elevated filling pressures and pulmonary congestion. Moderate or severe PAR after TAVI has become rare, however, even mild PAR may be associated with a significant increase in event rates compared with none/trace PAR. Important measures for reducing even small amounts of PAR are meticulous assessment of aortic root anatomy by CT and careful planning of the procedure including sizing of the prosthesis, adequate implantation technique (correct height, commissural alignment, etc.), and the technological improvements of the devices by manufacturers (such as the introduction of external sealing skirts). Some individual anatomies pose particular challenges, such as bicuspid valves, elliptic annuli, or severe annular calcifications. Assessment of PAR may be challenging. Currently, echocardiography, particularly, transoesophageal echocardiography (TOE) is preferred as the primary imaging method, with magnetic resonance recommended for equivocal patients. Treatment of PAR includes often post-dilatation with appropriately sized balloons or percutaneous closure of localized paravalvular leaks.

In another study, Meredith *et al*.^3^ investigated the effect of TAVI on LA function and geometry. They performed a systematic review and meta-analysis of 12 studies comprising 1066 patients that had echocardiographic data including LA speckle tracking available before and after TAVI. They report a significant reduction in indexed LA volume by 2.72 mL/m^2^ and an improvement in LA reservoir function by 3.71%. Six studies reported a small improvement in LA contractile function within 6 months of the TAVI, but not on long-term follow-up. In summary, this systematic review documents significant (albeit small) negative LA remodelling after TAVI, which is associated with improved distensibility, as measured by improved LA reservoir function.

Cardiac power output (CPO) is an echocardiography-derived measure of cardiac performance that incorporates both pressure and flow components and is calculated as: cardiac output × mean blood pressure/LV mass × 0.222 (conversion factor). Miyahara *et al*.^4^ explored the prognostic value of CPO in 794 consecutive patients undergoing TAVI for severe aortic stenosis with preserved LV ejection fraction (EF). CPO was measured by transthoracic echocardiography at discharge after TAVI. Patients in the lowest CPO tertile had significantly lower LV EF, significantly larger LA’s, and more patient-prosthesis mismatch. Over a follow-up of ∼2 years, CPO showed a dose-dependent association with outcomes, with the highest rate of the composite endpoint (death and HF hospitalizations) in the lowest tertile of CPO. On multivariable Cox regression analysis, CPO emerged as independent predictor of outcomes along with LV mass, LA volume, pulmonary artery pressure, and atrial fibrillation (AF). The data suggests that CPO may be a powerful measure to be incorporated with other clinical and echocardiographic variables into predictive models of mid-term outcomes after TAVI in patients with preserved EF.

As mentioned earlier, rheumatic valve disease is the predominant VHD aetiology in low- to middle-income countries, where it is responsible for over a million of premature deaths. De Azevedo Figueiredo *et al*.^5^ used two-dimensional LA speckle tracking to assess LA function in 493 patients with moderate to severe rheumatic mitral stenosis (mean valve area = 1.1 cm^2^). Using conditional inference tree analysis validated through sample splitting, including other potential variables such as age, LA size, and LV function, LA reservoir strain emerged as the single most important predictor of new-onset AF. On follow-up of 3.8 years, age, LA reservoir strain, tricuspid regurgitation (TR) severity, net atrioventricular compliance, and valve intervention were the most important predictors of adverse clinical outcomes (death or mitral valve surgery), with LA reservoir strain being the main predictive variable in patients aged ≤49 years.

Finally, the tricuspid valve—after being ‘the forgotten valve’ for decades—has recently received increasing attention. Significant TR is observed in 0.55% of the general population, is secondary (functional) in 90% of patients, and is an independent predictor of outcome in HF patients. Surgical treatment for isolated TR carries a high risk, therefore transcatheter repair and replacement therapies have recently raised widespread interest. In their review article, Mazzola *et al*.^6^ present a step-by-step protocol for echocardiography in the context of tricuspid transcatheter edge-to-edge repair. Transthoracic echocardiography is central in the initial morphological assessment of the tricuspid valve, in quantification of TR severity, and in evaluation of left and right ventricular function, and pulmonary congestion. TOE, on the other hand, provides a detailed evaluation of valve morphology and TR mechanism, and is the principal method for determining patient eligibility for edge-to-edge repair. Once eligibility is established, TOE plays a central role for guidance of the procedure: Often- deep-oesophageal or transgastric views including 3D imaging offer the best window for assessment of the tricuspid valve. Careful TOE imaging is the key to ensure proper positioning and clocking of the device, ascertain appropriate grasping of the leaflets, and document reductions in TR after device deployment.

In a small observational study with 77 patients, Zancanaro *et al*.^7^ assessed the short-term clinical outcomes of transcatheter tricuspid valve repair or replacement (almost half of the patients underwent percutaneous direct tricuspid annuloplasty) compared with conventional medical therapy. On 1-year follow-up, patients in the transcatheter treatment arm (*n* = 26) had significantly better clinical outcomes with regard to HF hospitalizations and overall death, and reported symptomatic improvements with a reduction in NYHA class and improvements in right-sided HF symptoms. Although neither randomized nor matched for differences in baseline characteristics, this study provides further support for the increasing clinical role of transcatheter therapies for TR.

## Data Availability

The data underlying this article are available in the European Heart Journal - Imaging Methods and Practice, at https://academic.oup.com/ehjimp. The datasets were derived from sources in the public domain: see references below.

## Lead author biography

Prof. Gaemperli studied medicine in Zurich, Switzerland, and specialized in internal medicine and cardiology in Switzerland and London, UK. He has trained in non-invasive cardiovascular imaging and interventional cardiology and has a strong research background in both fields. He has served as the vice president of the European Association of Cardiovascular Imaging from 2016–18 and is currently the president of the Swiss Working Group for Interventional Cardiology. He currently holds a position of consultant interventional cardiologist at the HeartClinic Hirslanden, Zurich, Switzerland.
